# Sintering Behavior of Magnesium-Substituted Fluorapatite Powders Prepared by Hydrothermal Method

**DOI:** 10.1155/2011/453759

**Published:** 2011-03-15

**Authors:** S. Nasr, K. Bouzouita

**Affiliations:** U.R. Matériaux Inorganiques, Institut Préparatoire aux Etudes d'Ingénieur, University of Monastir, Rue Eben El Jazar 5019, Monastir 5000, Tunisia

## Abstract

Magnesium-substituted fluorapatite powders were synthesized by hydrothermal method, and their sintering behavior was investigated by dilatometry in the temperature range 25–1100°C. Analysis of the obtained powders by X-ray diffraction and ^31^P NMR spectroscopy showed that the powders consisted of a single apatite phase and no amorphous phase has been formed. Compared to pure fluorapatite, the shrinkage of the substituted samples occurred in two steps and the temperature at which the sintering rate was maximum is lower. In addition, the shrinkage was interrupted by an expansion of the samples due to the formation of a liquid phase. The latter phase led to the crystallization of needle-crystals by a dissolution-diffusion-reprecipitation process.

## 1. Introduction

Owing to their physicochemical and biological properties, the hydroxyapatite (Ca_10_(PO_4_)_6_(OH)_2_, HA) and to a lesser extent the fluorapatite (Ca_10_(PO_4_)_6_F_2_, FA) have been extensively investigated over the last three decades. They offer important opportunities for applications in a diversity of areas particularly in medicine and dentistry [1–7]. Moreover, many applications have become possible thanks to their notable stability and their ability to accommodate a great number of substitutions; both cationic and anionic substitutions are possible [8–10]. For example, Ca^2+^ ion can be substituted by various divalent cations such as Mg^2+^, Sr^2+^, Pb^2+^, Cd^2+^, and so forth. Among these ions, some of them (Sr^2+^, Cd^2+^ or Pb^2+^) lead to a solid solution in the whole range of composition [11–13], while the incorporation of other ions such as Mg^2+^ or Mn^2+^, into the apatite structure remains limited [14–19]. 

Magnesium, whose concentration varies from 0.44 to 1.23 wt% [20], is one of the most abundant elements that are present in hard tissues. It has a prominent effect on the osteoporosis [21] and mineralization process [22, 23]. Thus, the synthesis of hydroxyapatite and fluorapatite powders in controlled Magnesium content is of a practical importance to develop biomaterials whose chemical composition is as close as possible to that of bone. Despite numerous studies that have been devoted to the incorporation of Mg into the hydroxyapatite structure [14–16], its substitution limit is a subject of controversy and the process of its incorporation remains unclear. Indeed, according to the used synthesis method, the substitution limit of this element in the hydroxyapatite varies from ~0.3 to 28.4 wt% [16, 24]. Furthermore, it is reported that all of the Mg used was not really incorporated into the apatite structure, but a significant proportion is adsorbed on the surface of the particles in an amorphous phase and/or in another form [14, 25].

Different methods can be used for synthesis the apatite such as solid-state reaction [26], coprecipitation [15, 17], sol-gel [27] and hydrothermal synthesis [18]. Hydrothermal synthesis offers a relatively simple and effective way to prepare Well-crystallized powders. Furthermore, samples with highly homogeneous composition and uniform size can be prepared by this method [28–30]. 

In a earlier work, we reported the synthesis of the Magnesium-substituted fluorapatites with the general formula of Ca_10−*x*_Mg_*x*_(PO_4_)_6_F_2_ (M_*x*_FA) (0 ≤ *x* ≤ 6) by the hydrothermal method and showed that the substitution limit is of about *x* = 2.5, (6.3 wt%) [18]; it is higher than that obtained by using the precipitation method [17]. The aim of this present study is to investigate the sintering behavior of these materials with *x* = 0, 1, and 2. After characterization by X-ray diffraction (XRD), nuclear magnetic resonance (MAS-NMR), transmission electronic microscopy (TEM), scanning electronic microscopy (SEM), and by a measure of the specific surface area, the as-synthesized powders have been submitted to a dilatometric study. The obtained ceramics have been investigated by XRD, ^31^P MAS-NMR, and SEM.

## 2. Experimental Procedure

### 2.1. Powders Preparation

Analytical grades Ca(NO_3_)_2_·4H_2_O, Mg(NO_3_)_2_·6H_2_O, (NH_4_)_2_HPO_4_, and NH_4_F weighed according to the stoichiometric formula of Ca_10−*x*_Mg_*x*_(PO_4_)_6_F_2_ with *x* = 0, 1, and 2, were dissolved into 5 cm^3^ of deionized water under vigorous stirring. Then, the pH value of the solution was adjusted to 9 using ammonia. After that, the solution was transferred into a Teflon-lined stainless autoclave (model 4749 Par Instrument). The autoclave was oven heated at 180°C for 6 h and, then, cooled to room temperature naturally. The collected precipitates were washed with deionized water and finally dried at 70°C overnight.

### 2.2. Sintering

The dilatometric curves were recorded with a Setaram TMA 92 dilatometer using the as-prepared powders. Pellets of 10 mm in diameter and 5 mm in thickness were uniaxially pressed and used as samples. A heating rate of 10°C/min was employed. 

### 2.3. Characterization

The (Ca + Mg)/P molar ratios in the as-prepared powders were evaluated by chemical analysis [31, 32]. Fluoride content was measured using a fluoride selective electrode (Ingold, PF4-L). XRD patterns of as-prepared powders and sintering samples were collected on a Philips X-pert diffractometer operating with Cu-K*α* radiation for a 2*θ* range from 20° to 60° with a step size of 0.02° and a counting time of 1 s per step. The phases in the as-prepared and sintering samples were identified by comparing the experimental XRD patterns to the standards compiled by the Joint Committee on Powder Diffraction and Standards (JCPDS cards). The average crystalline size, *D*_XRD_, was determined by means of the Scherrer's formula

(1) $$D_{\text{XRD}}=\frac{0.9\lambda }{\beta \cos\;\theta },$$

where *λ* is the X-ray wavelength of the monochromatic X-ray beam, *β* is the full width at half maximum, and *θ* is the peak diffraction angle. 

The diffraction peak corresponding to the (002) Miller plane family of FA was chosen to estimate the crystal size along the *c* crystallographic axis.

The MAS-NMR analysis was conducted on ^31^P nuclei using a Brucker 300 WB spectrometer at a resonance frequency of 121.5 MHz. Spinning rate of the sample at the magic angle was 8 kHz. ^31^P chemical shift was referenced to an external standard of an aqueous solution of 85% H_3_PO_4_.

The specific surface area (SSA) of the as-synthesized powders was measured with a Belsorp 28 SP apparatus using the BET method, while nitrogen was used as an adsorbed gas. The particle size, *D*_BET_, was estimated by assuming the primary particles to be spherical

(2) $$D_{\text{BET}}=\frac{6}{\rho s},$$

where *ρ* is the theoretical density of the powder and *s* is the SSA.

The particle size of the powders was analysed by laser granulometry (Malvern instruments, Mastersizer 2000). 

Transmission electron microscopy investigation was carried out on a Philips CM 200 microscope. The TEM specimen was prepared by dispersing the powder sample in acetone and dropping the dispersion on a carbon film supported on a copper grid.

Scanning electron microscopy (PHILIPS SEM, Model XL 30) was used to observe the particle size of the as-synthesized powders and the microstructure of the sintered samples.

## 3. Results and Discussion

### 3.1. As-Prepared Powders

The chemical analysis results for the as-prepared powders are presented in Table 1. The (Ca + Mg)/P molar ratios were close to the theoretical value of 1.67 for the fluorapatite while the fluoride content was close to that in the nominal composition. 

**Figure 1:**
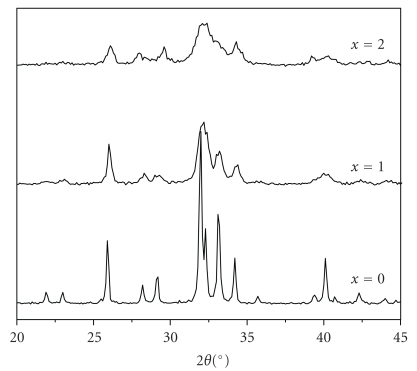
X-ray powder diffraction patterns of M_*x*_FA samples.

As it is shown in Figure 1, all the peaks correspond to an apatite phase, which is consistent with the JCPDS # 00-071-0880 file data for FA (space group P6_3_/m). There was no evidence of any crystalline phase other than the apatite phase. However, the presence of impurities in an amorphous phase or in small quantities could not be excluded, especially since the formation of such phases has been evoked during the synthesis of Mg-substituted HA [14] and FA [17], respectively. Notice that the patterns contained no halo. As it can be seen from the XRD patterns, the 2*θ* values of the M_*x*_FA samples slightly shift towards higher angles compared to the pure FA (Figure 1). This peak shift consecutive to a lattice contraction was caused by the Mg substitution for Ca, Mg^2+^ (coord. 6: *r*_Mg^2+^_ = 0.72 Å) being smaller in size compared to Ca^2+^ (coord. 6: *r*_Ca^2+^_ = 1.00 Å) [33]. The lattice contraction testifies that Mg was incorporated into the FA framework [18]. 

**Figure 2:**
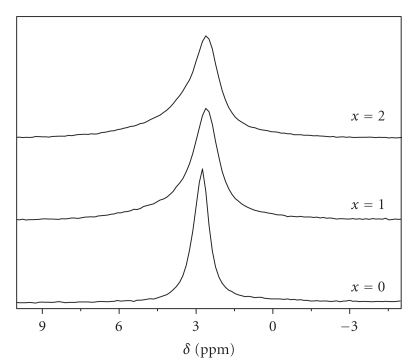
^31^P MAS NMR spectra of M_*x*_FA powders.

In order to find evidence to the presence of Mg in another phosphate phase than the apatite, the samples were submitted to MAS-NMR analysis. Indeed, in most cases, the X-ray diffraction permits the observation of crystalline phases, while the solid state NMR allows the characterization of the local atomic environments in both crystalline and amorphous phases.  Figure 2 shows that all the ^31^P MAS NMR spectra exhibited a single-resonance peak, which is characteristic of phosphorus in an apatite environment. For FA, the chemical shift is of 2.75 ppm. This value is in agreement with the values reported in the literature for FA [34]. However, for the substituted samples, a slight chemical shift towards lower values occurred as the Mg concentration increased, and concomitantly the peaks became broader. The broadening, which was also observed in the XRD data, was due to the reduction of the particle size and the crystallographic disorder, induced by the difference between the ionic radii of both cations Ca^2+^ and Mg^2+^. A computer *deconvolution* of the signals for *x* = 1 and 2 revealed two peaks corresponding to two different phosphate environments, one intense at* 2.75 *attributed to the phosphate groups in close proximity in Ca^2+^ ions, and a small peak at around 3.73 ppm, it could be related to the phosphate groups in close proximity in Mg^2+^ ions. These results showed that the phosphorus environment undergoes little change during the incorporation of Mg. In agreement with this, the FTIR spectra of the as-synthesized powders showed only the characteristic bands of PO_4_ group in an apatitic environment [18]. Thus, it seems that no amorphous phase was present in the powders, and if Mg was assumed to be adsorbed on the particle surface, as it is generally reported for HA and FA [14, 25], it should take place in another form that cannot be detected either by XRD or NMR. 

As it can be seen from the Table 2, the increase in Mg content in term of Mg/Mg+Ca ratio from 0.0 to 0.2 resulted in a decrease in crystallite size and an increase in BET SSA. This is in line with the inhibitor role of Mg on the apatite crystallization in solution [14–17]. Assuming the powder particles to be spherical in shape, the average sizes were calculated using (2) (Table 2). For the nonsubstituted FA, the value of *D*_BET_ was similar to that of *D*_XRD_ (Table 2). However, for the substituted samples, the values were slightly larger. This difference was probably due to the presence of agglomerates or to the role of Mg on the apatite crystallisation as it is indicated above, causing the broadening of the diffraction peaks. In order to understand the role of agglomerates, their size was determined by dynamic laser scattering method.

**Figure 3:**
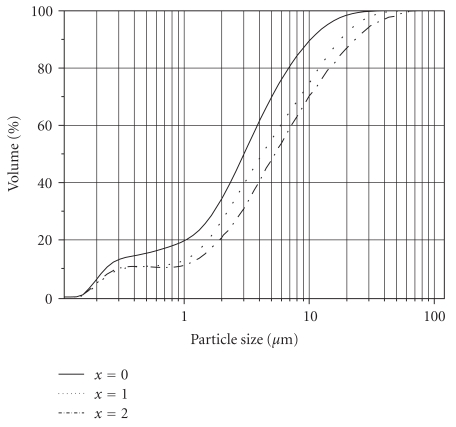
Particle size distribution of the as-synthesized powders.

Figure 3 shows the particle size distributions of the powders. Regarding this figure, the particle size distributions of powders were bimodal: the first particle population had a narrow size distribution 0.12–0.60 *μ*m while the particles corresponding to the second population were distributed in a wider size range 0.60–60 *μ*m. Notice that the particle sizes of the substituted FA shifted towards the high values with the increase of the Mg content. The mean agglomerate sizes *D*_50_ of the powders are given in Table 2, too. These values are much higher than those of *D*_XRD_ or *D*_BET_. 

**Figure 4:**
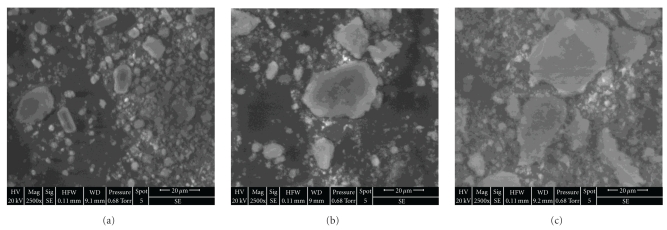
SEM micrographs of M_*x*_FA samples: (a) *x* = 0, (b) *x* = 1, and (c) *x* = 2.

**Figure 5:**
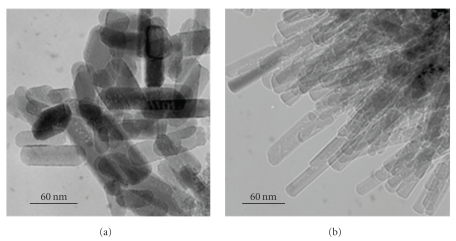
TEM micrographs of M_*x*_FA samples: (a) *x* = 0 and (b) *x* = 1.

In agreement with the particle size distributions, the SEM micrographs showed that the powders were composed of two populations (Figures 4(a)–4(c)), and as it can be observed, these Figures confirmed also that the degree of agglomeration increased with the increase of the Mg content. The morphology and size of the powder crystallites were also investigated by transmission electron microscopy.  Figure 5 shows the TEM images of M_*x*_FA samples for *x* = 0 and 1. As it is observed in Figure 5(a), the FA crystallites were needle-like with length of about 95 ± 15 nm and width of about 26 ± 2 nm. From the Figure 5(b), there is not too much difference in morphology. For the M_1_FA sample, the crystallite size decreased to 76 ± 10 nm and 18 ± 1 nm, respectively. Also, the powders in both samples seemed to be agglomerated corroborating the results of the scanning electron microscopy and the analysis by the laser granulometry. The agglomerates formation could be related to the agglomeration of particles with the submicrometer sizes (first population) consecutive to the increase of attraction between them with a decreasing particle size, due to Van der Waals interaction. The formed agglomerates continued to agglomerate until they reached the micrometer sizes (second population) [35–37].

### 3.2. Sintering Behavior

**Figure 6:**
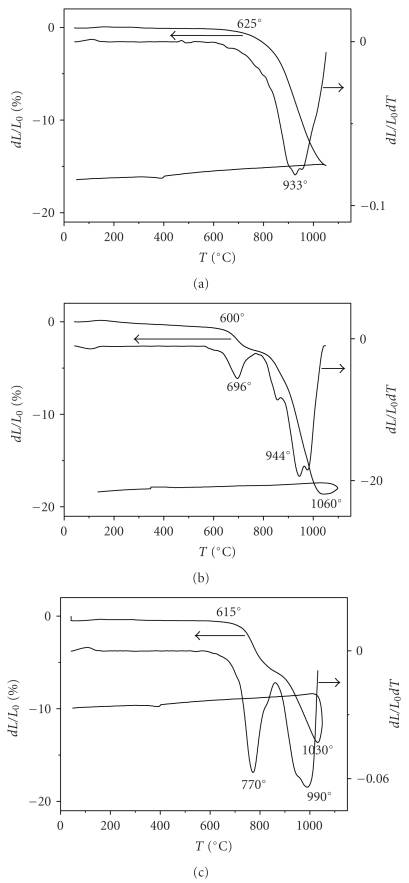
Shrinkage and shrinkage rate of M_*x*_FA samples as a function of temperature: (a) *x* = 0, (b) *x* = 1, and (c) *x* = 2.

The sintering behavior of the M_*x*_FA powders was investigated from room temperature to 1100°C at a heating rate of 10°C/min in the air atmosphere.  Figure 6 displays the shrinkage curves versus the temperature and the corresponding shrinkage rate curves of FA, M_1_FA, and M_2_FA samples. A comparative study between FA and M_*x*_FA powders shows a difference of behavior. For FA, the shrinkage occurring in one step begging at about 625°C and its maximum rate occurred at 933°C. The overall linear shrinkage after heating until 1050°C was approximately 15%. Compared with the powder prepared by the precipitation method [38], the temperature at which the shrinkage onset of the FA synthesized by the hydrothermal method commenced and the temperature at which the sintering rate was maximum were much lower. This behavior would be related to the characteristics of the powders synthesized by the hydrothermal method: small particle size and large specific surface area. As reported by Hidouri et al. [25], the shrinkage of substituted samples occurred in two steps with a shift of its beginning to the lower temperatures. A Such behavior was also observed for the Mg-substituted hydroxyapatite [39]. The first one starting from 600°C and 615°C and achieving a maximum at 696°C and 770°C for M_1_FA and M_2_FA, respectively, was probably caused by a particle rearrangement further to a better crystallisation of the powder. Indeed, it is well known that the incorporation of Mg into the apatite framework induces a decrease of the powders crystallinity [14–18]. The second step, corresponding to the major densification reached a maximum rate at about 944°C and 990°C for M_1_FA and M_2_FA, respectively. However, for the latter material, the shrinkage was interrupted by an expansion occurring from 1030°C. M_1_FA also presented an expansion, but after the end of the densification and its amplitude was much lower. For the M_1_FA material, the overall shrinkage was of 19%. Compared to M_1_FA, the shift to higher values of the shrinkage beginning temperature and that at which the densification rate was maximum for M_2_FA would be attributed to the more important of degree agglomeration of the powder. In the same way, we can explain the more important relative shrinkage of the first step. The expansion was probably due to the formation of a liquid phase. Such a phase was previously observed during the heat treatment of FA [38] and M_1_FA [25]; it resulted from a binary eutectic between apatite and CaF_2_ contained in the starting powder such as an impurity [25, 38, 40]. In the case of FA, the liquid phase is formed at 1205°C [40], while for the substituted samples, its formation occurred at lower temperatures; this shift towards the low temperatures is probably due to the presence of Mg.

**Figure 7:**
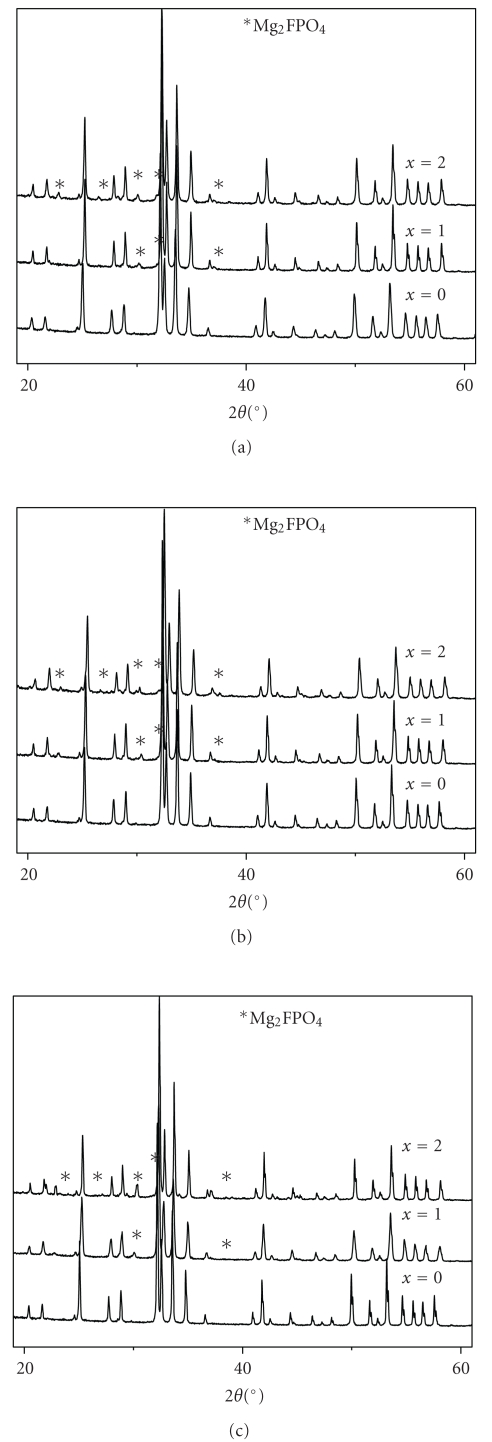
X-ray diffraction patterns of M_*x*_FA samples sintered at various temperatures for 1 h: (a) 950°C, (b) 1050°C, and (c) 1200°C.

After sintering, XRD analysis revealed, besides the apatite phase, the presence of a secondary phase corresponding to the wagnerite, Mg_2_FPO_4_ (JCPDS #00-074-1236) (Figures 7(a)–7(c)). It crystallizes in the monoclinic system (space group P2_1_/c) [41]. According to Hidouri et al. [25], this phase, which crystallizes from 650°C would result from an amorphous phase. Nevertheless, as indicated above the MAS-NMR analysis of the as-synthesized powders did not revealed the presence of any another environment for the phosphorous than that of the apatite phase. Also, the XRD patterns did not indicate the presence of an amorphous phase in the powders. Moreover, it was reported that the content of Mg_2_FPO_4_ formed depends on the quantity of magnesium used rather than the heat treatment temperature [25]. In order to check the accuracy of this assumption, an estimation of the relative proportion MFA/Mg_2_FPO_4_ was conducted on the XRD data using the Fullprof program [42]. The proportions of both compounds determined from the XRD patterns of samples sintered between 950 and 1200°C are presented in Table 3. As it can be seen, the formation of Mg_2_FPO_4_ depended on the temperature rather than the Mg amount incorporated into the apatite structure. Indeed, for a given *x* value, the proportion of Mg_2_FPO_4_ increased with the temperature. However, at a given temperature this proportion slightly varied with the concentration of Mg.

**Figure 8:**
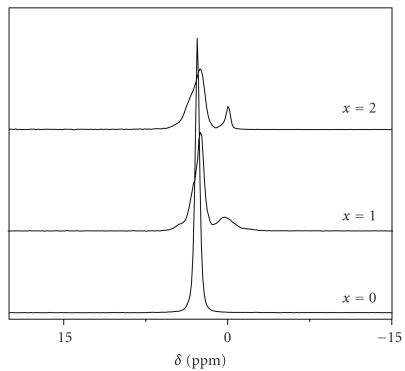
^31^P MAS NMR spectra of M_*x*_FA samples sintered at 1000°C for 1 h.

Figure 8 shows typical ^31^P MAS NMR spectra of samples sintered at 1000°C for 1 h. The spectrum of the unsubstituted FA had one resonance, compared to that of the as-prepared sample; it is sharper and intense, due to the improve of the crystallinity of the powder. This confirms that the FA was not affected by the sintering. In contrast, the spectra of the substituted samples were different from those of the as-prepared samples, indicating that structural changes have occurred during the sintering, in agreement with the results of the XRD analysis. As mentioned above, Mg_2_FPO_4_ crystallizes in the monoclinic system with the space group P2_1_/c, and in this case, the phosphorus presents four crystallographic sites [43]. In agreement with this, we should observe four resonances for this latter phase. However, the spectra exhibited in addition to that of the apatite phase two peaks of very low intensity at 4.4 and 0.3 ppm, respectively, and a shoulder at 3.1 ppm. Taasti et al. studying by the ^31^P MAS-NMR technique Zn_2_F(PO_4_), which has the wagnerite structure, observed also only three resonances [44].

**Figure 9:**
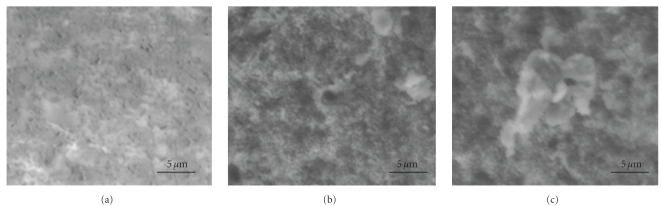
SEM micrographs of M_*x*_FA samples sintered at 950°C for 1 h: (a) *x* = 0, (b) *x* = 1, and (c) *x* = 2.

**Figure 10:**
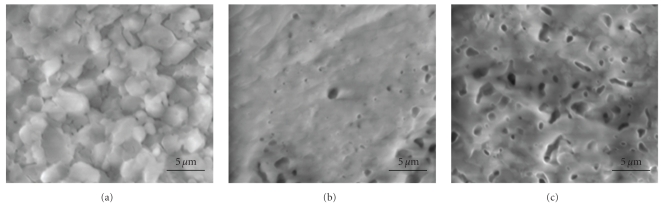
SEM micrographs of M_*x*_FA samples sintered at 1050°C for 1 h: (a) *x* = 0, (b) *x* = 1, and (c) *x* = 2.

**Figure 11:**
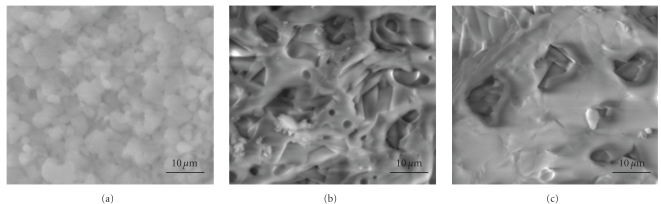
SEM micrographs of M_*x*_FA samples sintered at 1200°C for 1 h: (a) *x* = 0, (b) *x* = 1, and (c) *x* = 2.

A microstructural study was performed using SEM. Typical microstructure of samples sintered at 950, 1050, and 1200°C for 1 h are given in Figures 9, 10, and 11, respectively. The microstructure of the sintered substituted samples was obviously different from the sintered nonsubstituted ones. In connection with the low sintered density, at 950°C the microstructure of all the sintered samples was characterized by a high porosity and the presence of small grains (Figure 9). The increase of the sintering temperature was accompanied by an increase in mean grain size. 

At 1050°C, the Figure 10(a) shows that the microstructure of nonsubstituted sample was constituted by distinct grains in the range from 3 to 4.7 *μ*m in size. Only some residual pores located at the triple points were observed. Notice that the grains were well crystallized. However, for the substituted samples, the number of the pores increased with the increasing of the Mg content and it is difficult to distinguish the grains boundary (Figures 10(b) and 10(c)). This porosity would prevent the densification. Such a microstructure could be due to the presence of the liquid phase.

At 1200°C, the pure FA fully densified exhibited equiaxed grains, with broad size of 6–8 *μ*m (Figure 11(a)). While, the micrographs 11(b) and 11(c) for substituted samples show a significant porosity, needle-crystals were observed within the pores. 

To promote the densification of some materials, a liquid phase is formed into the microstructure of the material using appropriate sintering aids. Several mechanisms have been proposed to explain the densification of these materials. But in general, the densification is divided into three distinct stages: (i) particle rearrangement after the formation of the liquid phase, (ii) solution-diffusion-precipitation of particles through the liquid phase, and (iii) solid-phase sintering after disappearance of the liquid phase [45, 46]. In the present study, the liquid phase formation was probably due as indicated above to the eutectic between the fluorapatite and the fluorine, which was present in the as-synthesized powder as impurity. In the absence of the liquid phase for both materials, the densification mechanisms during sintering can occur by lattice and/or grain boundary diffusion. While for the substituted samples, in the presence of the liquid phase a dissolution-diffusion-reprecipitation process occurred. However, the densification was hindered by the crystallization of the needle-crystals. This recrystallization of the material could be facilitated by the high solubility of apatite in the liquid phase formed. Thus, after dissolution of the apatite up to saturation, there was crystallization of needle-crystals; such a microstructure was observed for Magnesium-substituted fluorapatite sintered with additives [47].

## 4. Conclusion

This work is devoted to the sintering behavior of Magnesium-substituted fluorapatites synthesized by hydrothermal method. The obtained and sintered powders were characterized by XRD, ^31^P MAS-NMR, SSA measurements, TEM and SEM. The results can be summarized as follows.

1. The as-prepared powders were needle-like nanoparticles and were a single crystallographic phase. They had a high specific surface area, which increased with the increasing of the Mg content, while the values obtained by the precipitation method [48] are much lower than those obtained by the hydrothermal method. However, agglomerates were formed during the synthesis. The agglomeration degree of powders presented the same trend as Mg.
2. The dilatometric study on powders without calcination showed that the shrinkage occurred in one step for pure FA and in two steps for the substituted samples. For these latter materials, the shrinkage was interrupted by an expansion whose amplitude increased with the increasing amount of Mg. In addition, the incorporation of Mg within FA lowered the temperature at which the highest densification rate was observed. The results obtained by Hidouri et al. [25] for the Mg-substituted fluorapatite, and Cacciotti et al. [39] for Mg-substituted hydroxyapatite showed the same shape of shrinkage curves as that obtained in this work.
3. The characterization of the sintered substituted samples showed the presence of a secondary phase corresponding to the wagnerite, M_g2_FPO_4_, whose content increased with the temperature. At high temperature, a liquid phase was formed and the formed amount increased with increasing Mg content. When this liquid phase was sufficiently abundant, there was crystallization of needle-crystals.

## Figures and Tables

**Table 1:** Chemical analysis data for as-prepared samples.

Nominal composition	Weight percent				Ca+Mg/P
	Ca	Mg	P	F	Molar ratio
Ca_10_(PO_4_)_6_F_2_	39.8	—	18.3	3.93	1.675
Ca_9_Mg_1_(PO_4_)_6_F_2_	36.3	2.44	18.5	3.73	1.668
Ca_8_Mg_2_(PO_4_)_6_F_2_	33.3	4.97	18.7	3.77	1.669

**Table 2:** Characteristics of as-prepared samples.

Nominal composition	Surface	*D*_(BET)_	*D*_(002)_	*D*_(50%)_
	(m^2^/g)	nm	nm	nm
Ca_10_(PO_4_)_6_F_2_	45.0	41.6	42.6	2900
Ca_9_Mg_1_(PO_4_)_6_F_2_	50.9	36.5	31.5	4100
Ca_8_Mg_2_(PO_4_)_6_F_2_	78.4	23.9	18.7	5200

**Table 3:** Phase composition of sintered samples (XRD data).

Nominal composition	Phase composition, wt%		
	950°C	1050°C	1200°C
Ca_10_(PO_4_)_6_F_2_	100 FA	100 FA	100 FA
Ca_9_Mg_1_(PO_4_)_6_F_2_	91.17 M_1_FA + 8.83 Mg_2_FPO_4_	90.91 M_1_FA + 9.09 Mg_2_FPO_4_	86.51 M_1_FA + 13.49 Mg_2_FPO_4_
Ca_8_Mg_2_(PO_4_)_6_F_2_	90.54 M_2_FA + 9.46 Mg_2_FPO_4_	90.49 M_2_FA + 9.52 Mg_2_FPO_4_	84.02 M_2_FA + 15.98 Mg_2_FPO_4_
